# Seroepidemiology and associated risk factors of *Toxoplasma gondii* in sheep and goats in Southwestern Ethiopia

**DOI:** 10.1186/s12917-016-0906-2

**Published:** 2016-12-09

**Authors:** Dechassa Tegegne, Amin kelifa, Mukarim Abdurahaman, Moti Yohannes

**Affiliations:** Department of Veterinary Microbiology and Public Health, College of Agriculture and Veterinary Medicine, School of Veterinary medicine, Jimma University, PO. Box 307, Jimma, Ethiopia

**Keywords:** Goat, Sheep, Toxo-latex, *T. gondii*, Seroprevalence, Southwestern Ethiopia

## Abstract

**Background:**

*T.gondii* is a global zoonotic disease and is considered as the most neglected tropical disease in sub-Saharan countries. The exact seroepidemiological distribution and risk factors for the infection of food animals and humans in Ethiopia was less studied although, such studies are important. The objective of the current study was to determine the seroprevalence and potential risk factors of *T. gondii* infection in sheep and goats in Southwestern Ethiopia.

**Methods:**

Cross sectional study was conducted from November 2014 to March 2015 in South west Ethiopia in four selected districts of Jimma zone (*n* = 368). Slide agglutination test (Toxo-latex) was used to detect anti-*T.gondii* antibodies. Logistic regression was used to determine potential risk factors.

**Results:**

An overall seroprevalence of 57.60% (212/368; 95% CI: 52.55–62.6) was detected. 58.18% (148/252; 95% CI: 52.75–64.88) and 55.18% (64/116; 95% CI: 46.13–64.23) sero prevalence was found in sheep and goats respectively. Multivariable logistic regression analysis showed that the risk of *T. gondii* infection was significantly higher in adult sheep and goats [(sheep: Odds Ratio (OR) = 2.5, confidence interval (CI): 1.19–5.23; *p* = 0.015), (goats: OR = 3.9, confidence interval (CI):1.64–9.41: *p* = 0.002)] than in young sheep and goats, in female [(sheep: OR = 1.93, CI: 1.11–3.36, *p* = 0.018, (goats: OR = 2.9, CI: 121–6.93, *p* = 0.002)] than in males sheep and goats, in Highland [(sheep: OR = 4.57, CI: 1.75–12.66, *P* = 0.000, (goats: OR = 4.4, CI: 1.75–13.66, *p* = 0.004)] than sheep and goats from lowland.

**Conclusion:**

This study indicates that seroprevalence of latent toxoplasmosis in small ruminants is high, therefore, it is decidedly indispensable to minimize risk factors exposing to the infection like consumption of raw meat as source of infection for humans.

**Electronic supplementary material:**

The online version of this article (doi:10.1186/s12917-016-0906-2) contains supplementary material, which is available to authorized users.

## Background

Toxoplasmosis is zoonotic disease caused by an obligate intracellular parasite known as *T. gondii* [1]. It is the most prevalent parasitic infections in human and veterinary medicine and has negative impacts on public health and animal production. *T. gondii* is believed to be the most triumphant parasitic pathogen in large scale [1]. Despite having adverse health effects analogous to those of salmonellosis and campylobacteriosis, toxoplasmosis is still a neglected and underreported parasitic infection. Human vaccines are not available and the results of the usage of the current anti-parasitic therapies are quite disappointing [2].

Toxoplasmosis is found globally; almost one third of the human population [1, 3]. The occurrence of toxoplasmosis has been significantly increasing as a result of the opportunistic infection of immune compromised patients, for instance, acquired immune deficiency syndrome (AIDS). In these people deaths usually result from rupture of cysts that lead to continued multiplication of tachyzoites [4]. Hence, encephalitis was presented as the main clinical manifestation of toxoplasmosis in AIDS patients as a result of reactivation of latent infection [5]. Majority of ocular cases at the present are associated with acquired toxoplasmosis, thus preventive strategies should be focused not only on pregnant women but also in the general population [2].

From wide range of farm animals, sheep and goats are more commonly infected with *T.gondii* than cattle and chicken. This parasite causes abortion and neonatal death in major monetary losses to sheep, goat and pig farming [3, 5]. This is more serious especially when primary infection occurs during pregnancy [6].

For evaluating the comparative significance of wide causes of toxoplasmosi*s* in human’s epidemiological survey still remains the main important approach. There have been a wide range of serological surveys conducted in different countries to determine the prevalence of toxoplasmosis in farm animals and humans; from North and South America [7–13], Europe [14–16], Africa [17–23] Asia [24, 25]. According to Australian Centre for International Agricultural Research (ACIAR) [26] *T.gondii* is extensively spread among farm animals and human with variable seroprevalence rates of 11–61% in goats, less than 10% in cows, 35–73% in cats, 75% in dogs, 11–36% in pigs, and 35–73% in humans.

In Africa different reports indicate widespread occurrence of toxoplasmosis. Thirty percent infection rate of toxoplasmosis was reported in goats in Botswana [20]. Limited studies have been carried out to investigate the magnitude of toxoplasmosis in animals and humans in Ethiopia so far. A preliminary serological study made in sheep and goat population around Nazareth showed an overall seroprevalence of 54.7% in sheep and 26.7% in goats using Enzyme linked Immunosorbent Assay (ELISA) and Modified Agglutination Test (MDAT) [21]. In another seroprevalence study of human toxoplasmosis of workers at Addis Ababa abattoir [22], reported a prevalence of 96.8% using an indirect haemagglutination assay [16], 80.7% in HIV patients in Agaro Health Centre in Jimma Zone [27] on top of this 6.6, 22.9 and 11.6% prevalence was reported in cattle, sheep and goats in central Ethiopia respectively [17].

In general, there is a scarcity of data on sero- epidemiology of toxoplasmosis in animals and humans in Ethiopia though numerous literatures associate human toxoplasmosis with utilization of raw/undercooked meat products of animal origin. The exact seroepidemiological distribution and risk factors for the infection of food animals in Jimma are unknown but, such studies are indispensable because consumption of raw meat is a popular tradition in Jimma. Thus, human toxoplasmosis in Jimma might have strong linkage with seroprevalence of the infection in food animals on top of this little is known about seroprevalence of *T.gondii* infection in sheep and goat Jimma zone. Therefore, the present study was intended with objective of estimating the seroprevalence and risk factors of *T.gondii* infection in small ruminants in Jimma zone.

## Methods

### Study areas

The study was carried out in four districts of Jimma zone namely Seka Chokorsa 20 km in Southeast, Mena 22 km in North east, Kersa 18 km in Northwest and Goma 50 km west of the Jimma. Jimma is a capital city of of Jimma zone which is located in Oromia regional state found 352 km away from the capital city (Addis Ababa) in the southwest Ethiopia. It is located at latitude of 7°13′– 8°56’ N and longitude of 35°52′–37°37’ E, and at an altitude ranging from 880 m to 3360 m above sea level (masl). The study areas were purposively selected to represent three agro-ecological zones such as highland (≥2300, masl), midland (1500–2300 masl) and lowlands (≤1500 masl). The area receives about 1530 mm rainfall that comes from the long and short rainy seasons. The mean temperature yearly ranges from 25 to 30 °C and 7 to 12 °C [28] (Additional file 1: Figure S1).

As it was reported by Central Statistical Agency census [29], the total population of Jimma zone is 2,642,114, from these Jimma town populations accounts 177,900 (Ethiopian statistical agency 2015 projected from 2007 census), 49.7 and 49.3% females and males respectively). From the total population in the zone, 2,204,225 (88.66%) are rural community engaged in agricultural activities for their livelihood. Jimma zone is potential source of livestock which contribute to the country growth and domestic production; about 466,154 of sheep, 194,677 of goats, 1,718,284 of cattle, 40,555 of donkeys, 30,541 of mules and 74,774 of horses [30].

### Study animals

The study subjects were sheep and goats. Small ruminant production in the study areas was mainly characterized by traditional and extensive type of management system. Mainly male’s sheep and goats are known to be kept for mutton production in most parts of the country while females are for breeding. The study dealt with animals kept by peasants in four districts of Jimma zone.

### Study design and sample size

Cross-sectional study design was used. Different age and sex groups of sheep and goats were included for this study. The study was conducted from November 2014 to March 2015. Serological investigation was used to detect anti-*T.gondii* antibodies from blood serum collected from sheep and goats in the districts under study. Since there was no previous expected prevalence in the area, sample size was calculated as it is stated by Thrusfield [31] using an expected prevalence of 50.0% a desired precision of 5% and with 95% level of confidence. Hence, the sample size was 384. Basically due to the fact that goat’s population at study area was very limited in number, therefore, only 116 goats and 252 of sheep sera were subjected to analyses, totally 368 samples were analyzed. Simple random sampling technique was carried out to collect sera from small ruminants.

### Blood collection

Sheep and goats was aseptically bled (approximately 5 ml) from the jugular vein by using 10 ml vacutainer tubes which contained no anti-coagulants or preservatives and vein puncture needle and needle holder was used and properly labelled with water proof marker with the necessary information. Blood sample was transported to Jimma University, microbiology and veterinary public health laboratory and was kept overnight at room temperature and then centrifuged at 3000 rpm for 10 min to get serum. The serum was collected in 1.5 ml Eppendorf tubes and kept at −20 °C until serologically tested for the presence of anti *T. gondii* antibodies.

### Serological examination

*T. gondii* antibodies were detected by the Toxo Latex slide Agglutination test following the procedure described by manufacturer SPINREACTGirona/Spain. Briefly, 50 μL sera samples was placed on toxo-latex agglutination slide and one drop of each positive and negative control into separate circles on the slide test and mixed thoroughly. 25 μL of toxo-latex reagent was added to 50 μL sera samples into separates circles and mixed thoroughly with stirrer then the mixture was spread over entire circle. The slide was placed on mechanical rotator at 96 rpm for 4 min. The presence or absence of visible precipitation was macroscopically examined immediately after removing the slide from the rotator. The formation of precipitation was recorded as positive and it indicates an antibody concentration equal or greater than 4 IU/ml.

### Data analysis

Data were recorded and coded using Microsoft Excel spreadsheet and analysed using SPSS version 20. Seroprevalence was calculated by dividing the number of animals possessing anti-*T.gondii* antibodies against the total number of animals tested. Relationship of risk factors with dependent variable was primarily assessed using cross tabulation. Univariable logistic regression analysis was performed and strength of association between risk factors and *T. gondii* infection were evaluated using odds ratios (OR). The 95% confidence interval (CI) and a significance level of α = 0.05 were used.

## Result

### Overall seroprevalence

Out of 368 animals examined 212 (57.60%, 95% CI: 52.55–62.65) were seropositive for *T.gondii* antibody; 58.8% (148/252; 95% CI: 52.75–64.88) and 55.18% (64/116; 95% CI: 46.13–64.23) seroprevalence was found in sheep and goats respectively. Both serum samples from sheep and goats showed positive reactions of similar proportions (Table 1). Their variation is statistically insignificant (*P* > 0.05).

**Table 1:** Seroprevalence of *T. gondii* antibody and logistic regression analysis of risk factors for sheep

Risk factors	Total	No of positive	Univariable		Multivariable	
Species^a^			COR (95% CI)	*P*-Value	AOR (95% CI)	*P*-Value
Sheep	252	148 (58.73%)	-		-	
Goat	116	64 (55.18%)	-		-	
Total	368	212 (57.60%)				
Age						
Young (<1 year)	40	15 (37.5%)	1		1	
Adult (>1 year)	212	133 (62.7%)	2.8 (1.39,5.63)	0.004	2.5 (1.19,5.23)	0.015
Sex						
Male	99	46 (46.5%)	1			
Female	153	102 (66.7%)	2.3 (1.37, 3.87)	0.002	1.93 (1.11,3.36)	0.018
Altitude						
Highland	113	79 (69.9.9%)	5.07 (2.23,1163)	0.000	4.57 (1.75,12.66)	0.000
Midland	104	58 (55.8%)	2.75 (1.22,6.19)	0.015	2.8 (1.23,6.54)	0.014
Lowland	35	11 (31.4%)	1			-

*COR* crude odd ratio, *AOR* adjusted odd ratio, *CI* Confidence Interval

^a^Species, *P* = 0.213

### Risk factors

Results of logistic regression analysis indicates that the potential risk factors related to sex, age and altitude revealed that the likelihood of *T. gondii* infection was higher in adult sheep (OR = 2.5), female sheep (OR = 1.95) and highland (OR = 4.57) when compared with male, young and lowland sheep respectively (Table 1). On top of this, the likelihood of *T. gondii* infection was higher in adult goats (OR = 2.5), female goats (OR = 1.95) and highland (OR = 4.4) when compared with male, young and lowland goats, respectively (Table 2).

**Table 2:** Seroprevalence of *T.gondii* and logistic regression analysis of risk factors for goats

Risk factors	Total	No of positive	Univariable		Multivariable	
			COR (95% CI)	*P*-value	AOR (95% CI)	*P*-Value
Age						
Young (<1 year)	40	14 (35%)				
Adult (>1 year)	76	50 (65.8%)	3.57 (1.59,7.98)	0.002	3.9 (1.64,9.41)	0.002
Sex						
Male	42	18 (42.9%)	1			
Female	74	46 (62.2%)	2.19 (1.01, 4.73)	0.035	2.9 (1.21,6.93)	0.002
Altitude						
Highland	59	38 (67.9%	4.52 (57,13.5)	0.005	4.4 (1.75,15.5)	0.004
Midland	38	19 (50%)	2.14 (0.714,6.43)	0.174	3.9 (1.64,9.41)	0.10
Lowland	22	7 (31.8%)	1			-

*COR* crude odd ratio, *AOR* adjusted odd ratio, *CI* Confidence Interval

## Discussion

Out of 368 animals examined the seroprevalence of anti-*T.gondii* antibody was found 212 (57.60%). This is comparable to the previous report from Ethiopia [32]. Different studies revealed that the prevalence from 0 to 100% was recorded in different areas of the world [33]. This difference in prevalence is depending up on cat density, climate condition, age of the animals, species, sex, altitude and management of animal production [3, 5, 34].

Lower prevalence values of 3.8, 4.3, 11.2, 12.1 and 16.9% were recorded by Sharma [35] in India, Samra [36] in South Africa, Ramzan [37] in Pakistan, Dubey and Foreyt [38] in the North America and Márcia de Figueiredo [12] in Brazil respectively. The current study estimated seroprevalence of *T.gondii* antibody in sheep was 58.73% and this is in agreement with the finding of 56.00% [32] in Central Ethiopia and higher seroprevalence in sheep has been reported when compared to the present finding [3, 12, 39, 40].

The seroprevalence of *T.gondii* antibody in goats was 55.18% is closely related to the finding of 59.4% from Egypt [41], but it is higher than the finding of 19.70 and 37.20% reported by Zewdu *et al*. [39, 42] and Yibeltal, [43] in Central Ethiopia and in South Wollo respectively. It is also higher than the 39, 31.7, 28.9, 27. 9 and 25.4%, reported from Thailand [44], Pakistan [37] and Brazil [12, 13, 23] respectively. In contrast, the prevalence of the present study is lower than the 67.9% reported from Zimbabwe [45]. The variations in the overall prevalence observed in the current study and the above studies could be due to differences in the access of small ruminants to contaminated feed and water, the climatic variation and the diagnostic techniques used [34, 46].

The present study indicated that sheep from the highland (OR = 4.57, CI: 1.75–12.66) and midland (OR = 2.8, CI: 1.23–6.54) areas of Southwest Ethiopia have significantly (*P* = 0.000) high risk of infection of *T.gondii* than those of from the lowland. Similarly, goats from the highland (OR = 4.4, CI: 1.75–15.5) and midland (OR = 3.9, CI: 1.64–9.41) areas of Southwest Ethiopia have significantly higher risk of *T. gondii* infection than those from the lowland (*P* = 0.004). This study was in agreement with the finding of 46.91% in highland and 46.24% in midland but lower prevalence in lowland 13.36% [39, 42] in Central Ethiopia. This variation among risk factors can be described by the variation in temperature and moisture in these areas. It is well known that the epidemiology of toxoplasmosis is influenced by the environment [3, 34]. Humidity increases, the chance of oocyst survival in the environment, thereby contributing to the higher seroprevalence. A dry climate has an impact on the survival and epidemiological distribution of the parasite [1, 47].

Correspondingly, seroprevalence of *T.gondii* antibody was high in adults (62.7.4% sheep and 65.8% goats) than in young animals (37.5.09% sheep and 35% goats). Statistically significant variation was observed among them. This finding is relatively similar to [48, 49] that reported prevalence (10.00%) in young and (26.76%) in adult. Multivariable logistic regression analysis showed that the likelihood of acquiring infection was higher in adult [(sheep: OR = 2.5, CI: 1.19–5.23; *P* = 0.015), (goats: OR = 3.9, CI: 1.64–9.41: *p* = 0.002)] than in young sheep and goats. Seroprevalence of *T.gondii* antibody increases with age in both species. This could be attributed to the reality that increment of the disease prevalence in older animals is due to exposure of animals to the risk factors for longer period of time than the younger ones [1, 12, 13, 39, 42]. With regard to sex risk factor, the study showed that the seroprevalence of anti-*T.gondii* antibody is higher in females (60.9%) than in males (54.64%) in both species. The result was similar to the finding of Zewdu *et al*. [39, 42] in female (34.39%) and males (19.43%).

Multivariable logistic regression analysis revealed that the likelihood of acquiring infection was higher in females [(sheep: OR = 1.93, CI: 1.11–3.36, *p* = 0.018), (goats: OR = 2.9, CI: 121–6.93, *p* = 0.002)] than in males sheep and goats. A small number of male sheep and goats are kept for breeding whilst others are culled and sold. In addition, the stress of lactation and pregnancy due to hormonal difference lead to immune-suppression that may expose the female sheep and goats to toxoplasmosis [50].

## Conclusions

This study indicated that seroprevalence of latent toxoplasmosis in small ruminants is high. Therefore, it is particularly requisite providing health education about its public health importance and risk factors that expose to humans the infection such as consumption of raw meat of small ruminants.

## Additional files

Additional file 1: Figure S1. Map of study districts. (JPG 324 kb)
Additional file 2: Raw data of sheep and goats. (XLSX 17 kb)
