# Supplementary figures and images for: Seroepidemiology and associated risk factors of Toxoplasma gondii in sheep and goats in Southwestern Ethiopia
Source: BMC Vet Res. 2016 Dec 9;12:280. doi: 10.1186/s12917-016-0906-2 (PMC5148880; doi:10.1186/s12917-016-0906-2)

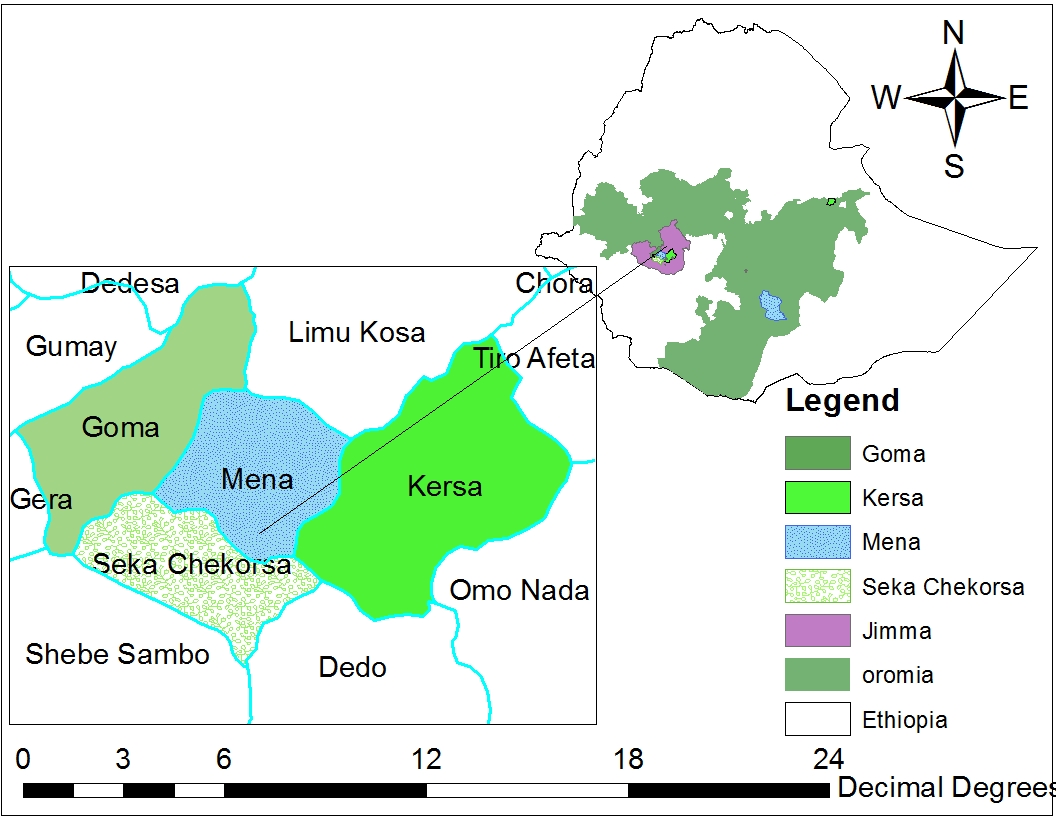

Supplement: Additional file 1: Figure S1. — Map of study districts. (JPG 324 kb) [file 12917_2016_906_MOESM1_ESM.jpg]
